# Spinal Cord Injury Secondary to “Head Banging” at a Punk Rock Concert

**DOI:** 10.7759/cureus.6707

**Published:** 2020-01-20

**Authors:** Hasanga Fernando, Joseph F Baker

**Affiliations:** 1 Orthopaedics, Faculty of Medical and Health Sciences, University of Auckland, Auckland, NZL; 2 Orthopaedic Surgery, Waikato Hospital, Hamilton, NZL

**Keywords:** spinal surgery, spinal cord injury, orthopaedics, head banging

## Abstract

Heavy metal and rock concert audience members may engage in "head banging." Despite various reports highlighting the potential risks involved, formal injury research in this area is limited. We report the first ever case of cervical disc prolapse resulting in cervical myelopathy secondary to head banging. Through this case, we aim to highlight the potential risks of head banging.

## Introduction

“Head banging” describes a violent and rhythmical movement of the head synchronous with the music being played [1]. It is a popular dance form among concert attendees and musicians, particularly at heavy metal concerts and music festivals. Head banging has its origin in 1968 when Led Zeppelin performed at the Boston Tea Party during their first tour of USA. The front row audience at the concert began head-banging in time with the music. Thus, the term “head-banger” to describe these participants was born following this incident. There are different styles of the head-banging motion such as the up-down, the circular swing, the full body or the side-to-side motion [1]. Although the practice of head banging is often associated with musical concerts, similar head movements can also be observed in certain Indian rituals [2].

There are various reports of head and neck injuries secondary to head banging in the literature [2-10]. However, formal injury research in this area is minimal. In this case report, we discuss a patient who presented with myelopathy secondary to a cervical disc prolapse due to head banging. An extremely rare complication, not found in the literature on the subject of injuries associated with head banging. We believe this will add to the growing body of knowledge in the area of neck injuries secondary to head banging.

## Case presentation

A 30-year-old man presents with acute onset of right arm weakness and intermittent paraesthesia, following an episode of “head banging” at a rock music concert. According to the patient’s own description, the “head-banging” motion involved rhythmical, repetitive and vigorous forward flexion and extension of the neck synchronous to the tune of the music. The head-banging episode lasted for a period of approximately two minutes before the inception of his symptoms. He also complained of “pins and needles” sensation travelling through his back on deep flexion or extension of the cervical spine. He denied any problems with fine motor function of the hands, balance or sphincter disturbance.

He had a 13-year history of attending various music concerts on a regular basis and frequently engaged in head banging. Ten years previously, he experienced a whiplash injury following a head-banging episode which resulted in two-day work leave due to the injury. However, he made a full recovery and was asymptomatic leading up to the current event.

On clinical examination, he was hyper-reflexic in both upper and lower extremities. He had a positive inverted radial reflex on the right side. In the upper limbs, weakness of the elbow extension was noted on the right side (4/5). In the lower limbs, there was a positive Babinski sign and clonus on the right side with approximately 10 to 15 beats. He had no overt sensory deficit to light touch. Romberg’s test was negative, and he was able to heel-toe walk satisfactorily. 

**Figure 1:**
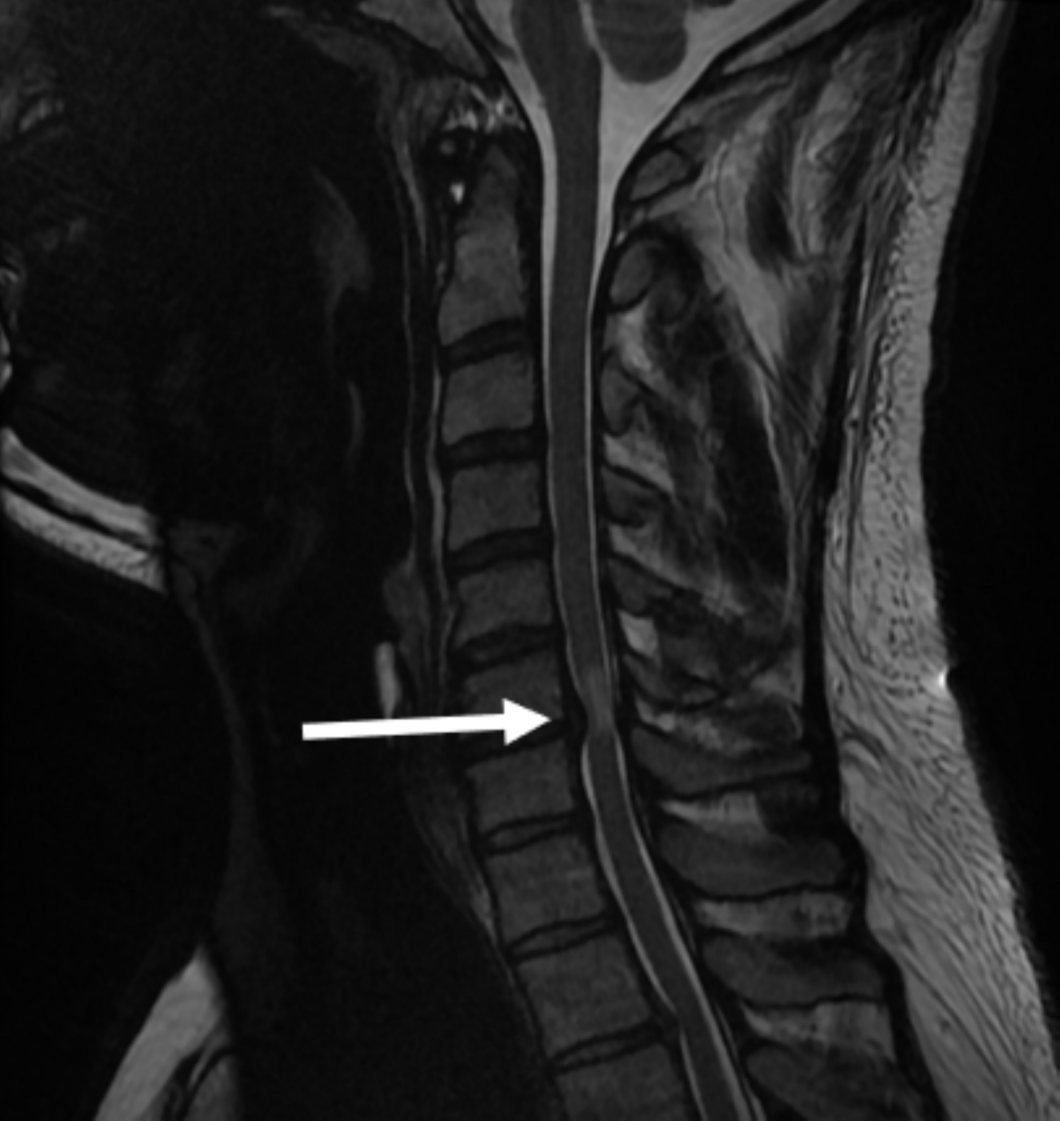
T2-weighted MRI showing spinal cord compression and focal cord oedema at the C6/7 level

On magnetic resonance imaging, a central disc protrusion at C6/7 level was noted with associated increased signal within the cord on T2-weighted images (Figure 1).

He underwent an ACDF (anterior cervical discectomy and fusion) via a left-sided approach. The right C6/7 foramen was directly decompressed. Reconstruction was performed using a polyetherethylketone cage packed with autologous iliac crest and plate. Day 1, following the operation, he was mobilising comfortably with resolution of radicular symptoms and improved power in the right upper extremity. His immediate and short-term recovery was uneventful.

At eight months following surgery, he has returned to work with no subjective features of radiculopathy or myelopathy. His Neck Disability Index was 14% (range is 0% [asymptomatic] to 100% [complete disablement]). Clinical examination reveals persistence of the inverted radial reflex on the right but resolution of the clonus in the right lower extremity. Plain radiographs demonstrated mature fusion across the disc space. Whilst still an avid concert-goer, he had refrained from further head banging.

## Discussion

The worldwide phenomenon of head banging is associated with various forms of injuries, especially head and neck injuries. It is often described as a violent activity that is popular among the audience of hard rock music and various subgenres of heavy metal music [1]. The patient in this case was listening to punk rock music at the time of the injury.

In 2005, doctors claimed that the guitarist, Terry Balsamo, from the band Evanescence, experienced a stroke due to head banging [1]. Based on the reports on this subject, musicians and concert attendees have an increased risk of injury secondary to head banging, due to the increased prevalence of this practice among this population. A myriad of injuries within this population are presented in the literature. These include several cases of subdural haemorrhages, a single case of traumatic aneurysm of the cervical vertebral artery, basilar artery thrombosis, carotid dissection and a mediastinal emphysema [3-11]. It is also important to note that injuries secondary to head banging can occur outside of a musical setting. There is a case of basilar artery thrombosis reported due to head banging as part of a South Indian religious ritual, and also two cases of bilateral mature cataracts in children with chronic head banging secondary to autistic spectrum disorder [2,12].

This current case is unique in representing the first known case of spinal cord injury as a result of head banging. Moreover, it is intriguing to consider whether there was an increased risk of cervical spinal injury from head banging in our patient due to the presence of relative canal stenosis. Fortunately, he recovered well with early surgical intervention. 

Whilst seemingly impossible to prevent head banging, Patton and McIntosh recommend limiting the neck range of motion during head banging and wearing protective equipment such as neck braces to limit the neck range of motion [1]. More prudently, this report should serve to alert clinicians of the potential for serious spinal injury secondary to head banging, effectively representing a self-inflicted injury. Neurological symptoms and signs with onset after this activity mandate thorough investigation.

## Conclusions

Head banging is a common dance form at certain classes of music concerts. With sudden deceleration forces dissipated along the spine, there is potential for serious spinal column injury as a result of head banging. Clinicians must remain alert to this possibility and thoroughly investigate symptoms and signs precipitated by this activity.
